# Typhoid Carriers

**Published:** 1908-03-28

**Authors:** 


### TYPHOID CARRIERS.

Although the subject of whether or not house-flies may be factors in the dissemination of many diseases, including typhoid fever, is a very interesting one, it is the purpose of the present article to deal with the question of human typhoid carriers only.

Persons who have suffered from typhoid fever, and who have recovered, may continue to be a source of infection to others in at least three ways. These are:—

1. By the persistence of purulent discharges containing typhoid bacilli long after the disappearance of the typhoid fever; for example, sinuses after operation for typhoidal empyema of the thorax, empyema of the gall-bladder, otitis media, and so forth.

2. By the persistence of typhoid bacilli in the urine after convalescence is well established.

3. By the persistence of typhoid bacilli in the stools. The first of these ways has been already mentioned and discussed in an article in this journal upon typhoidal pyelonephritis. The second way has been discussed at length elsewhere. It is with the third way in which a patient may be a typhoid carrier, namely, by persistence of the bacilli in the stools, that it is proposed to deal briefly here.

There has been comparatively little work done upon the length of time that typhoid bacilli may still be found living in the stools of patients who have recovered from typhoid fever. One of the reasons for this is, of course, the difficulty of the process of distinguishing between the bacillus coli communis and the bacillus typhosus, even by careful cultivations in special laboratories. When a patient has ceased to be ill, laborious and expensive bacteriological work upon his excreta have naturally seemed unnecessary. No book can tell one the precise point at which it is unnecessary, in ordinary practice, to continue special precautions about disinfecting the stools; and so it happens that disinfection is persisted with until the patient is fit to go away for a change of air; from that time onwards it has generally been assumed that no further precautions are necessary.

No doubt this is true in a great many cases, but it is not surprising to hear that it is by no means true in all. It has always been rather a mystery how sporadic cases of typhoid fever arise, when no trace of how the infection arose can be found. It is now beginning to appear that some such cases are really due to infection from perfectly healthy people who, having had typhoid fever formerly, are still carrying about with them, and emitting, living typhoid bacilli wherever they go.

The significance of this from a public health point of view is obvious, and a good deal of work is likely to be done upon the subject in the near future. The practical importance of the matter to individuals may be gathered from the following notice which appeared in the *Morning Post*:—

"Our Bristol correspondent states that for some time the authorities of Bentry Home for Inebriates, near Bristol, were troubled by a continuing outbreak of typhoid fever among those in the institution. From September 1906 to November 1907 there were 28 cases and two deaths, and the ordinary efforts failed to account for or stop the spread of the disease. Dr. D. S. Davies, the medical officer of health for the City of Bristol, took up the matter in November and made special pathological investigations in conjunction with the Medical Inspector of the Home Office, with the result that experience has been gained which is considered to be of great importance in connection with the disease. Dr. Davies reported to the Bristol Health Committee that, judged by quite recent pathological work in Germany, the case seemed to point to the existence of a 'carrier' case of chronic typhoid intermittently, but unconsciously, infecting the food or milk. A special pathological inquiry was instituted into the condition of certain inmates, and late in December it was proved that one of these—a woman who had the disease six years ago—was a typhoid 'carrier.' That person was isolated on November 13. The last case at the institution was on November 24, which was within the incubation period, but from then the institution has been free. Beyond the German cases one in New York, and one in Scotland, Dr. Davies stated that he was unaware of any other successful investigation of typhoid outbreaks on this line of research. Dr. Davies is going to the Home Office to discuss the matter with the Government officials."

It is to be hoped that the inquiry will show, in the first place, that typhoid carriers of six years' duration are extremely rare; and that, in the second place, a definite ruling will be obtained as to when the disinfection of the stools may be safely dispensed with in ordinary average cases.

